# Investigating the Potential of Full-Fat Soy as an Alternative Ingredient in the Manufacture of Low- and High-Moisture Meat Analogs

**DOI:** 10.3390/foods12051011

**Published:** 2023-02-27

**Authors:** Yung-Hee Jeon, Bon-Jae Gu, Gi-Hyung Ryu

**Affiliations:** Department of Food Science and Technology, Food and Feed Extrusion Research Center, Kongju National University, Yesan 32439, Republic of Korea

**Keywords:** extrusion, meat analogue, meat alternative, plant-based meat, extruded protein, high-moisture meat analog, textured vegetable protein, whole soy

## Abstract

The increase in meat consumption could adversely affect the environment. Thus, there is growing interest in meat analogs. Soy protein isolate is the most common primary material to produce low- and high-moisture meat analogs (LMMA and HMMA), and full-fat soy (FFS) is another promising ingredient for LMMA and HMMA. Therefore, in this study, LMMA and HMMA with FFS were manufactured, and then their physicochemical properties were investigated. The water holding capacity, springiness, and cohesiveness of LMMA decreased with increasing FFS contents, whereas the integrity index, chewiness, cutting strength, degree of texturization, DPPH free radical scavenging activity, and total phenolic content of LMMA increased when FFS contents increased. While the physical properties of HMMA decreased with the increasing FFS content, its DPPH free radical scavenging activity and total phenolic contents increased. In conclusion, when full-fat soy content increased from 0% to 30%, there was a positive influence on the fibrous structure of LMMA. On the other hand, the HMMA process requires additional research to improve the fibrous structure with FFS.

## 1. Introduction

The Food and Agriculture Organization of the United Nations (FAO) estimates that by 2030, per capita annual meat consumption will be 100 kg in industrialized countries [1]. Mass meat production induces ethical issues for animals, low land utilization, water usage, adverse environmental effects, etc [2]. Specifically, increasing meat products can boost carbon dioxide levels, increasing greenhouse gas formation [3,4]. Thus, consumers are starting to embrace plant-based meat analogs as an alternative source of real meat products because they are more environmentally-friendly and sustainable as a nutrient of protein as well as an aspect of animal welfare and animal rights [5,6,7].

At present, there are many technologies for producing meat analogs, such as extrusion cooking [8,9,10], freeze structuring [11], electrospinning [12], mechanical elongation [13], in vitro cultured meat [14], and the shear-cell technique [15]. Among the technologies, extrusion cooking is one of the most representative methods for the manufacture of meat analogs by using high temperatures, shear, and pressure [16,17,18]. Extrusion cooking is widely used for manufacturing many food products, such as puffed snacks, pasta products, noodles, breakfast cereals, meat analogs, gelatinized starch, dough, baby food, and others, because of its benefits of flexibility, low cost, high output, and quality control [8,10].

Extrusion cooking is classified into low- and high-moisture processing types, according to the degree of addition of moisture content into the extruder and the presence of a cooling die at the end of barrels. The low-moisture extrusion process for manufacturing meat analogs produces expanded meat analogs, and the expansion phenomenon causes sponge-like structures for the meat analogs [19]. Low-moisture meat analog (LMMA) requires hydration prior to making patties, chunks, and nuggets [20]. On the other hand, high-moisture extrusion cooking can produce non-expanded meat analogs that have denser and highly fibrous structures by using a cooling die [19]. More complex formulations are possible with high-moisture extrusion cooking, and it is not necessary to use highly soluble ingredients, making it a more economical technology [21,22].

Meat analogs, produced by extrusion cooking, are mainly composed of 50–95% (dry basis) plant-based proteins [23]. The most widely used plant-based protein in producing meat analogs using extrusion processing is soy protein due to its gelation, functional, and nutritional properties [22]. There are many types of soy protein in the meat analog market based on protein contents and extract processes, such as soy protein isolate (SPI), soy protein concentrate (SPC), full-fat soy (FFS), defatted soy flour (DSF), etc. [5]. Among them, SPI is the one mainly used in many research projects and industries for manufacturing plant-based meat analogs. In the extraction step for SPI, many steps are required to obtain concentrated and purified proteins, which cause environmental pollution and are expensive. In contrast, full-fat soy (FFS) is not only rich in nutrients such as complex carbohydrates, soluble fibers, and isoflavones but it is also easily accessible and cost-effective due to its minimal processing requirements [24,25].

Most research on extruded meat analogs (LMMA and HMMA) has centered around the use of SPI-based formulas due to their functional properties and smooth extrusion process for manufacturing [26,27]. Conversely, the impact of the manufacturing process on the texturization of FFS has not been explored, despite its potential as a substitute for SPI, due to the difficulties associated with its extrusion process. In this study, we aimed to fill this gap by investigating the texturization of FFS using two types of extrusion methods and examining the physical and antioxidant properties of LMMA and HMMA.

## 2. Materials and Methods

### 2.1. Materials

Soy protein isolate (SPI), full-fat soy (FFS), wheat gluten (WG), and corn starch (CS) were purchased from Plant Albumen Co., Ltd. (Pingdingshan, China), Korea Seed & Variety Service (Jecheon, Republic of Korea), Roquette Frères (Lestrem, France), and Samyang Co. (Ulsan, Republic of Korea), respectively. The crude protein and fat contents of SPI (84.87 ± 0.1% and 1.84 ± 0.2%), FFS (38.60 ± 0.3% and 20.07 ± 0.1%), and WG (77.81 ± 0.3% and 0.24 ± 0.2%) were measured using the Dumas method [28] and Danlami et al. [29], respectively. The formulation of raw materials for producing LMMA and HMMA is shown in Table 1.

### 2.2. Manufacturing of Meat Analogs by Low- and High-Moisture Extrusion Cooking

**Figure 1:**
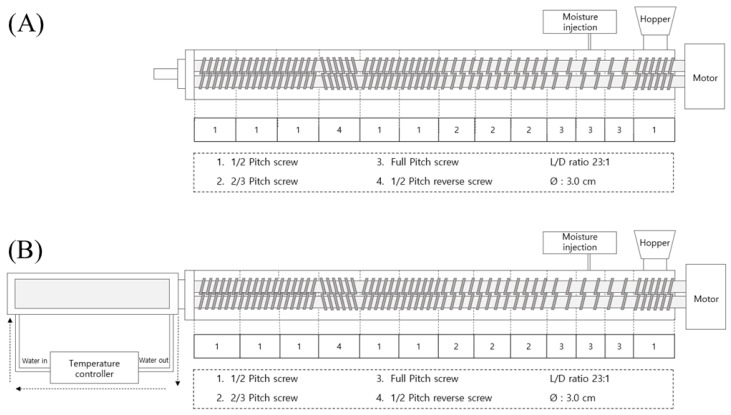
Low- (**A**) and high-moisture (**B**) extrusion processes and screw configurations used in this experiment.

Low- and high-moisture extrusion cookings were performed using a co-rotating intermeshing twin screw extruder (THK31T-No.5, Incheon Machinery Co., Incheon, Republic of Korea) with a 3 cm screw diameter and a 69 cm length (L/D: 23:1). The extrusion conditions—40% feed moisture, 160 °C barrel temperature, 250 rpm screw speed, and a slit die with dimensions of 1 cm (W) × 0.45 cm (H) × 8 cm (L)—were set for the low-moisture extrusion process to manufacture LMMA (Figure 1A). High-moisture extrusion cooking for HMMA was performed with 60% feed moisture, 160 °C barrel temperature, 150 rpm screw speed, and a long cooling die (dimensions of 7 cm (W) × 1 cm (H) × 50 cm (L)) cooled by 20 °C water with a water circulator (Duksan Cotran Co., Ltd., Daegu, Republic of Korea) (Figure 1B). At least 30 extrudates were collected for each sample after the condition was stable. The extrusion process for LMMA was performed until 30% of the FFS inclusion level because of the collapse of the texturized structure at the FFS level above 40%.

After the extrusion cooking process, the extruded samples were cut into 1 cm × 1 cm pieces for subsequent texture profile analysis and to determine the cutting strength of both low-moisture meat analogs (LMMA) and high-moisture meat analogs (HMMA). The cut LMMAs were dried at 50 °C for 12 h and stored at room temperature, and the cut HMMAs were stored in a −18 °C freezer and thawed in a 4 °C refrigerator for 24 h (FR-S690FXB, Klasse Auto Co., Ltd., Seoul, Republic of Korea) before the following analysis. Chemical properties were measured by using the dried LMMA and freeze-dried HMMA samples as ground samples (50–70 mesh particles).

### 2.3. Texture Profile Analysis and Cutting Strength

Texture profile analysis (TPA) was performed with a texture analyzer (Compac-100, Sun Science Co., Ltd., Tokyo, Japan) using a 2.5 cm diameter cylinder probe with a 10 kg maximum peak stress. The cutting strength (CS) was determined using a cutting probe (0.75 × 3.83 cm) with a 2 kg maximum peak stress. The LMMA sample was hydrated in a water bath at 70 °C for 1 h and then equilibrated at room temperature, and the HMMA sample was equilibrated at room temperature before the determination of the TPA and CS. The meat analogs for CS were cut along the vertical direction (transversal strength, F_v_) and parallel direction (longitudinal strength, F_p_) of the samples’ flow direction. The calculation of springiness, cohesiveness, chewiness, and CS was expressed according to the equation below [30]. The results were averaged from 6 measurements.

Springiness (%) = D_2_/D_1_ × 100 (1)

D_1_: distance reached for the first maximum stress

D_2_: distance reached for the second maximum stress

Cohesiveness (%) = A_2_/A_1_ × 100 (2)

A_1_: area underneath the first compression curve

A_2_: area underneath the second compression curve

Chewiness (g) = cohesiveness × springiness × highest peak force (g)(3)

Cutting strength (g/cm^2^) = highest peak force (g)/cross-sectional area (cm^2^) (4)

### 2.4. Water Holding Capacity

The water holding capacity (WHC) of LMMA and HMMA was determined by the modified methods of Gu and Ryu [31] and Diaz et al. [32], respectively. Approximately 5 g (on a dry basis) of samples were weighed. The LMMA was hydrated at 70 °C in a water bath for 1 h as per Gu and Ryu [31], and the HMMA was hydrated at 50 °C for 16 h as per Diaz et al. [32]. After that, the samples were drained for 15 min. WHC was expressed as grams of water retained per gram of dried sample using Equation (5).

Water holding capacity (g/g) = (weight of wet sample − weight of dry sample)/(weight of dry sample) × 100(5)

### 2.5. Degree of Texturization

Degree of texturization was calculated by Equation (6) [33].

Degree of texturization = F_v_/F_p_ (6)

where F_v_ is the transversal strength, and F_p_ is the longitudinal strength.

### 2.6. Integrity Index

The integrity index was determined by a modified method of Gu and Ryu [31]. A 5 g (dry basis) sample that was sunk in 100 mL of distilled water was autoclaved at 121 °C for 15 min, and then the sample was cooled and drained for 30 s. 100 mL of distilled water was added into the sample and homogenized at 17,450 rpm for 1 min, and then they were dried on a 20-mesh sieve at 105 °C for 12 h. The integrity index was calculated according to Equation (7).

Integrity index (%) = (weight of dry residue on sieve)/(weight of sample) × 100(7)

### 2.7. DPPH Free Radical Scavenging Activity

DPPH free radical scavenging activity was determined as per Brand-Williams et al. [34] with some modifications. A 2 g sample was extracted at room temperature by ethanol (80%) for 2 h, and then the extracted sample was centrifuged at 3000 rpm for 0.5 h. 3.9 mL of DPPH solution (0.0024 g DPPH reagent/100 mL of methanol) was mixed with the supernatant (0.1 mL). The absorbance (at 515 nm) of the solution was read after the mixture was incubated at room temperature in a dark room for 0.5 h. The DPPH free radical scavenging was calculated by Equation (8).

DPPH free radical scavenging activity (%) = (A_0_ − A)/A_0_ × 100 (8)

where A_0_ is the absorbance of the blank, and A is the absorbance of the sample.

### 2.8. Total Phenolic Contents

Total phenolic contents (TPC) were determined as per Slinkard and Singleton’s method [35]. A 2 g sample was extracted with 10 mL of ethanol (80%) for 2 h, and then the extract was centrifuged at 3000 rpm for 0.5 h. A 1.5 mL of 10% (*v/v*) Folin-Ciocalteu reagent was mixed with 0.3 mL supernatant for 5 min with a vortexer, and then the mixture was mixed with another solution (1.5 mL of Na_2_CO_3_ (60 g/L)) and incubated at room temperature for 2 h. After incubation, a wavelength of 765 nm was utilized to measure the absorbance of the solution. TPC was calculated as mg/g of gallic acid equivalents in milligrams per gram of dried sample (mg GAE/g).

### 2.9. Macrostructure

The macrostructure of cross-sectional LMMA and HMMA was observed after the samples were cut into 1 cm (W) × 1 cm (D). For the fibrous structure of LMMA and HMMA, the extruded samples (LMMA and HMMA) were cut into 3 cm (W) × 5 cm (D) and 5 cm (W) × 7 cm (D), respectively. The cut samples were opened in the longitudinal direction and then photographed for observation of the fibrous structure.

### 2.10. Statistical Analysis

All experiments were performed in triplicate unless otherwise stated and analyzed using SPSS (version 27.0, IBM-SPSS, Thornwood, NY, USA). Analysis of variance (ANOVA) and comparison of means were performed using Duncan’s multiple range tests at *p*  <  0.05. Correlation coefficients among the data were determined using Pearson’s correlation coefficient (*r*).

## 3. Results and Discussion

### 3.1. Texture Profile Analysis, Cutting Strength, and Water Holding Capacity

The textural properties (springiness, cohesiveness, and chewiness) and cutting strength of the vertical (V-CS) and parallel (P-CS) directions of LMMA and HMMA are summarized in Table 2. Springiness means how quickly an extruded meat analog is recovered after deformation by a probe; cohesiveness indicates the internal strength of bonds; and chewiness shows the energy requirement for masticating the food [36,37]. Significant differences in the springiness and cohesiveness of both LMMA and HMMA were observed, showing that higher FFS content in LMMA and HMMA caused a decrease in springiness and cohesiveness. Springiness is connected to protein content, and the addition of FFS decreases protein content comparatively, resulting in a decrease in protein cross-linking strength [36]. Ma and Ryu [38] also reported that the springiness and cohesiveness of meat analogs were mainly affected by cross-linking formation in the internal structure. The springiness of both LMMA and HMMA showed a positive correlation with cohesiveness (*r* = 0.987 and 0.990, respectively, *p* < 0.01) (Table 3 and Table 4), and the FFS caused a more negative effect on the springiness of LMMA than that of HMMA, resulting in 97.43 ± 3.4 (FFS 0%) to 79.32 ± 5.1% (FFS 30%) for LMMA and 93.03 ± 0.8 (FFS 0%) to 88.58 ± 1.2% (FFS 30%) for HMMA. The result could be due to the fact that high-moisture extrusion cooking is a more effective method of making fibrous structures compared to low-moisture extrusion cooking, maintaining molecular interactions in spite of higher FFS content [39]. The intermolecular protein cross-linking occurs in the cooling die, resulting in high-fibrous structures [40]. Additionally, increasing moisture content can increase the interactions between hydrogen bonds-disulfide bonds, and hydrophobic interactions-disulfide bonds [41].

The chewiness and cutting strength (in both vertical and parallel directions) of LMMA and HMMA had contrary results, showing that, with an increase in FFS content, those of LMMA were increased but those of HMMA were decreased. The increase in LMMA’s texture properties could be due to the interaction of lipids and proteins during the extrusion cooking process [42]. The reason for this is due to the reduction of the expansion ratio of LMMA with increasing FFS content due to the lubricant behavior of the lipids in FFS in the extruder barrel. Then, the behavior resulted in a decrease in the buildup of pressure for the vaporization of water, increasing both non-covalent and covalent interactions to build protein networks with suitable fibrous structures [43,44]. A water-phase change that causes the expansion phenomenon by the vaporization of water in the mixture of raw materials can increase the distance between the substances, resulting in weak structures in meat analogs [45,46]. On the other hand, the HMMA process, in general, did not involve the expansion phenomenon, and thus the vaporization of water by pressure drop did not occur during the process that extends between the protein, starch, and lipid molecules. Lipids are expected to interact with proteins with a saturation limit determined by the number of hydrophobic sites [41], but the decrease in the texture properties of HMMA might be due to the excessive lipid content that disturbs the molecular bonds during the extrusion cooking. According to Van Hoan et al. [47], a high moisture content can reduce the die pressure, thus reducing the amount of lipid lost during the extrusion. In other words, high-moisture extrusion cooking, which has a higher moisture content than low-moisture extrusion cooking with a low die pressure, could cause excessive lipid content that hinders molecular bonding. Alzagtat and Alli [48] reported that lipids might be coated on the surface of the protein aggregates, which prevented the protein molecules from cross-linking.

**Figure 2:**
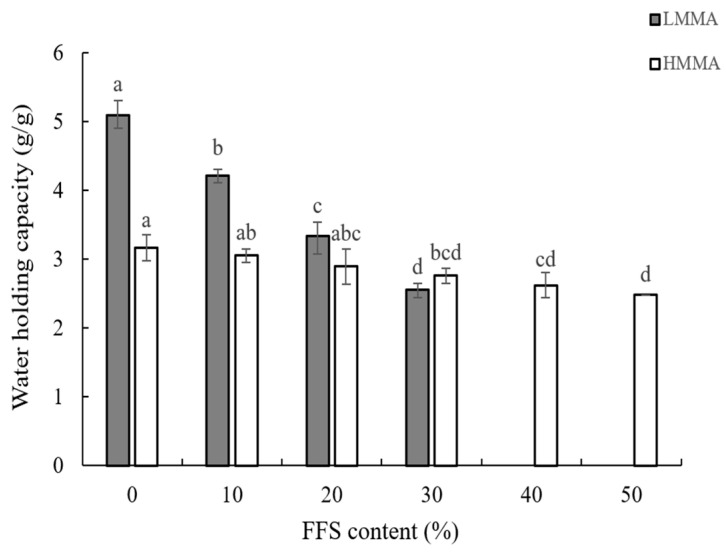
Water holding capacity of meat analogs with various full-fat soy (FFS) contents. LMMA: low-moisture meat analog; HMMA: high-moisture meat analog. Different letters (a–d) above the bars indicate the significantly different for each extrusion type (LMMA and HMMA) (*p* ≤ 0.05) by Duncan’s multiple range tests.

The water holding capacity (WHC) of LMMA and HMMA is shown in Figure 2. As shown, the lowest WHCs of 2.55 ± 0.1 g/g at FFS 30% for LMMA and 2.48 ± 0.0 g/g at FFS 50% for HMMA were observed, while the highest WHCs were 5.09 ± 0.21 g/g for LMMA and 3.16 ± 0.19 g/g for HMMA at FFS 0%. This is because the WHC is directly affected by the porosity of the meat analogs [49]. The empty spaces are filled by water that builds intra- and inter-hydrogen bonds caused by hydroxyl groups [50]. On the other hand, the reduction of porous structure could be due to the increase in lipid contents from FFS. Ottoboni et al. [51] reported that high lipid content might lead to a decrease in back-pressure during extrusion cooking, resulting in poor expansion.

WHC had positive correlations with springiness (*r* = 0.934, *p* < 0.01) and cohesiveness (*r* = 0.950, *p* < 0.01) of LMMA and negative correlations with chewiness (*r* = −0.910, *p* < 0.01), V-CS (*r* = −0.972, *p* < 0.01), and P-CS (*r* = −0.911, *p* < 0.01) (Table 3). It was consistent with the results of Gu and Ryu [52] that the WHC of LMMA is positively correlated with springiness and cohesiveness. In contrast, those of HMMA had positive correlations, springiness (*r* = 0.778, *p* < 0.01), cohesiveness (*r* = 0.764, *p* < 0.01), chewiness (*r* = 0.821, *p* < 0.01), V-CS (*r* = 0.807, *p* < 0.01), and P-CS (*r* = 0.767, *p* < 0.01) (Table 4).

### 3.2. Degree of Texturization and Integrity Index

The degree of texturization (DT) can be used as an indicator for fibrous structure formation [53], and the integrity index indicates the residue of meat analogs after hydrating, autoclaving, homogenizing, and drying [31,33]. The higher integrity index means the better the texturization, the higher the integrity index, since the meat analogs’ texture remained strong and unweakened after the harsh process steps [19,26,31,54]. Therefore, the DT and integrity index of LMMA and HMMA generally showed a positive correlation (*r* = 0.955 and *r* = 0.768, respectively) (*p* < 0.01) (Table 3 and Table 4) [26,55].

**Figure 3:**
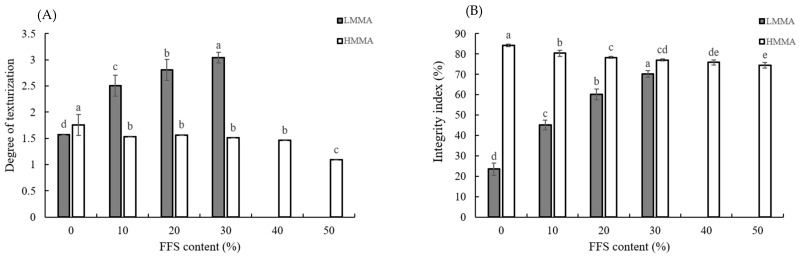
Degree of texturization (**A**) and integrity index (**B**) of extruded low- and high-moisture meat analogs with different full-fat soy (FFS) content. LMMA: low-moisture meat analog; HMMA: high-moisture meat analog. Different letters (a–e) above the bars indicate the significantly different for each extrusion type (LMMA and HMMA) (*p* ≤ 0.05) by Duncan’s multiple range tests.

With increasing FFS content from 0% to 30%, the DT and integrity index of LMMA increased, but those of HMMA decreased up to 50% of FFS content (Figure 3). The highest DT and integrity index were observed at LMMA with 30% FFS content (3.04 ± 0.1 and 70.28 ± 1.6%, respectively), and the lowest DT and integrity index were determined at HMMA with 50% FFS content (1.09 ± 0.0 and 74.46 ± 1.4%, respectively). The increase in DT and integrity index of LMMA could be a result of the interactions among phenolic compounds in FFS, protein, starch, and lipid molecules. Polyphenols and protein molecules are known to interact with each other reversibly (hydrogen bonding, hydrophobic bonding, and van der Waals forces) and irreversibly (covalent bonds) [27,53,56]. Alzagtat and Alli [47] also reported that lipids play a role as a plasticizer by forming complexes with starch, protein, and lipid during the extrusion cooking process, which can contribute to the stabilized fibrous structure. Conversely, the reason for the decrease in DT and integrity index of HMMA could be because an excessive amount of lipid hindered protein-protein, protein-starch, protein-lipid, and protein-polyphenol interactions during extrusion cooking.

### 3.3. DPPH Free Radical Scavenging Activity and Total Phenolic Contents

**Figure 4:**
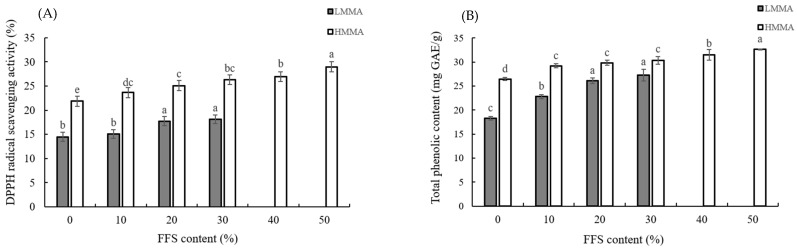
DPPH free radical scavenging activity (**A**) and total phenolic contents (**B**) of meat analogs with various by full-fat soy (FFS) contents. LMMA: low-moisture meat analog; HMMA: high-moisture meat analog. Different letters (a–e) above the bars indicate the significantly different for each extrusion type (LMMA and HMMA) (*p* ≤ 0.05) by Duncan’s multiple range tests.

DPPH free radical scavenging activity and total phenolic contents (TPC) were conducted to find out the antioxidant properties of meat analogs with FFS. A significant change in DPPH free radical scavenging activity and TPC of LMMA and HMMA was observed when FFS was added. The lowest values of DPPH free radical scavenging activity and TPC for both LMMA and HMMA were observed at FFS 0%, and DPPH free radical scavenging and TPC were increased by increasing FFS content from 0 to 30% for LMMA and 0 to 50% for HMMA (Figure 4). FFS content had a significant positive effect on the values of DPPH free radical scavenging activity and TPC that are related to antioxidant activity, showing the highest values of DPPH free radical scavenging activity were 18.14 ± 0.4% for LMMA and 28.99 ± 0.8% for HMMA [57]. This could be because FFS originally contained more phenolic compounds such as tannins, polyphenols, flavonoids, and phenolic terpenes compared to SPI [58]. Aludatt et al. [59] also reported that the phenolic content of the full-fat meal was higher than that of defatted ones, showing higher antioxidant activity.

### 3.4. Macrostructure

Digital photographs of LMMAs and HMMAs with different FFS content are shown in Table 5. The LMMA was not texturized when the FFS content was higher than 40%. In contrast, the HMMA was texturized to 50% FFS content. The fibrous structure is one of the key factors in judging whether meat analogs have a meat-like texture [60]. The fibrous structure of LMMA increased as the FFS content increased, and LMMA with a FFS content of 30% had the most fibrous structure and well-arranged layers than other LMMAs. The fibrous structure of HMMA was decreased by increasing the FFS content, but HMMA with an FFS of 50% did not exhibit definite layers or fibrous structure. The sponge-like structure of LMMA decreased with an increase in the FFS content, but HMMA showed a dense and layered structure. The structure of LMMA is due to the many pores caused by the expansion phenomenon that resulted from the pressure drop caused by the pressure difference between the inside and outside of the extruder. However, the HMMA was formed with a dense and fibrous structure because of the aggregation effect in the cooling die [26].

## 4. Conclusions

Overall, FFS was determined to be a promising ingredient for manufacturing meat analogs as an alternative source to SPI in this study. FFS content had a significant effect on the physical and antioxidant properties of LMMA and HMMA (*p* < 0.05). In addition, LMMA with 30% FFS content showed the most fibrous structures and the highest texture properties (chewiness, cutting strength, integrity index, and degree of texturization). However, texturization was not possible with over 40% FFS content in the LMMA process, but the HMMA process could manufacture texturized proteins with up to 50% FFS content, which completely replaced SPI in this study. The antioxidant properties of LMMA and HMMA increased as the FFS content increased. Further research is needed to increase FFS content for the LMMA process and enhance the texture properties of the HMMA by optimizing the independent process variables of the extrusion process. This study will contribute to enhancing the quality of meat analogs in terms of nutrition and texture aspects and removing the complex extraction steps for producing soy protein isolate, resulting in a reduction in cost and environmental pollution for the manufacture of meat analogs.

## Figures and Tables

**Table 1:** Formulation of extruded low- and high-moisture meat analog with different full-fat soy content.

Extrusion Type	Full-Fat Soy (%)	Soy Protein Isolate (%)	Wheat Gluten (%)	Corn Starch (%)
LMMA	0	50	40	10
	10	40	40	10
	20	30	40	10
	30	20	40	10
HMMA	0	50	40	10
	10	40	40	10
	20	30	40	10
	30	20	40	10
	40	10	40	10
	50	0	40	10

**Table 2:** Texture profile analysis and cutting strength (vertical and parallel) of meat analogs with various full-fat soy contents.

Extrusion Type	FFS Content (%)	Springiness (%)	Cohesiveness (%)	Chewiness (g)	Cutting Strength (g/cm^2^)	
					Vertical	Parallel
LMMA	0	97.43 ± 3.4 ^a^	94.15 ± 3.5 ^a^	263.42 ± 98.6 ^c^	551.3 ± 38.9 ^d^	351.2 ± 30.4 ^c^
	10	84.57 ± 1.3 ^b^	79.74 ± 2.3 ^b^	399.16 ± 82.2 ^b^	999.7 ± 69.3 ^c^	403.4 ± 64.7 ^bc^
	20	83.87 ± 5.3 ^bc^	74.95 ± 3.5 ^b^	436.72 ± 43.5 ^b^	1193.4 ± 68.2 ^b^	427.5 ± 45.1 ^b^
	30	79.32 ± 5.1 ^c^	67.58 ± 6.0 ^c^	537.67 ± 34.5 ^a^	1677.5 ± 66.2 ^a^	552.0 ± 31.1 ^a^
HMMA	0	93.03 ± 0.8 ^a^	79.07 ± 1.2 ^a^	4295.64 ± 130.9 ^a^	1236.8 ± 128.8 ^a^	711.7 ± 114.8 ^a^
	10	93.78 ± 0.6 ^a^	79.15 ± 0.7 ^a^	3967.45 ± 114.2 ^b^	1020.7 ± 52.8 ^b^	667.9 ± 30.1 ^ab^
	20	90.01 ± 1.0 ^b^	74.45 ± 2.0 ^b^	3559.24 ± 175.4 ^c^	977.7 ± 57.5 ^b^	625.5 ± 37.5 ^bc^
	30	88.58 ± 1.2 ^b^	73.11 ± 1.8 ^b^	3170.47 ± 172.5 ^d^	947.2 ± 63.8 ^b^	626.0 ± 41.6 ^bc^
	40	86.26 ± 1.5 ^c^	69.73 ± 4.1 ^c^	2550.08 ± 206.1 ^e^	821.1 ± 70.6 ^c^	562.3 ± 47.4 ^c^
	50	75.09 ± 3.6 ^d^	54.51 ± 2.8 ^d^	1183.40 ± 115.6 ^f^	477.9 ± 35.0 ^d^	438.6 ± 39.3 ^d^

Different letters (a–f) in the same column for each extrusion type indicate the significantly different (*p* ≤ 0.05) by Duncan’s multiple range tests. FFS: Full-fat soy content; LMMA: low-moisture meat analog; HMMA: high-moisture meat analog.

**Table 3:** Pearson correlation matrix of physical properties for low-moisture meat analogs.

	WHC	Springiness	Cohesiveness	Chewiness	V-CS	P-CS	DT	Integrity Index
WHC	1							
Springiness	0.934 **	1						
Cohesiveness	0.950 **	0.987 **	1					
Chewiness	−0.910 **	−0.905 **	−0.917 **	1				
V-CS	−0.972 **	−0.949 **	−0.961 **	0.928 **	1			
P-CS	−0.911 **	−0.875 **	−0.891 **	0.917 **	0.968 **	1		
DT	−0.925 **	−0.964 **	−0.957 **	0.841 **	0.910 **	0.790 **	1	
Integrity index	−0.962 **	−0.949 **	−0.952 **	0.866 **	0.965 **	0.881 **	0.955 **	1

Water holding capacity (WHC); vertical cutting strength (V-CS); parallel cutting strength (P-CS); degree of texturization (DT). Values with ** were significantly different (*p* < 0.01).

**Table 4:** Pearson correlation matrix of physical properties for high-moisture meat analogs.

	WHC	Springiness	Cohesiveness	Chewiness	V-CS	P-CS	DT	Integrity Index
WHC	1							
Springiness	0.778 **	1						
Cohesiveness	0.764 **	0.990 **	1					
Chewiness	0.821 **	0.976 **	0.964 **	1				
V-CS	0.807 **	0.916 **	0.900 **	0.950 **	1			
P-CS	0.767 **	0.864 **	0.840 **	0.889 **	0.958 **	1		
DT	0.715 **	0.889 **	0.882 **	0.907 **	0.902 **	0.752 **	1	
Integrity index	0.777 **	0.749 **	0.739 **	0.837 **	0.809 **	0.720 **	0.768 **	1

Water holding capacity (WHC); vertical cutting strength (V-CS); parallel cutting strength (P-CS); degree of texturization (DT). Values with ** were significantly different (*p* < 0.01).

**Table 5:** Fibrous and cross-sectional structures of extruded high-moisture meat analogs with different full-fat soy (FFS) content.

Extrusion Types	Structure	Full-Fat Soy Content (%)					
		0	10	20	30	40	50
LMMA	Cross-sectional	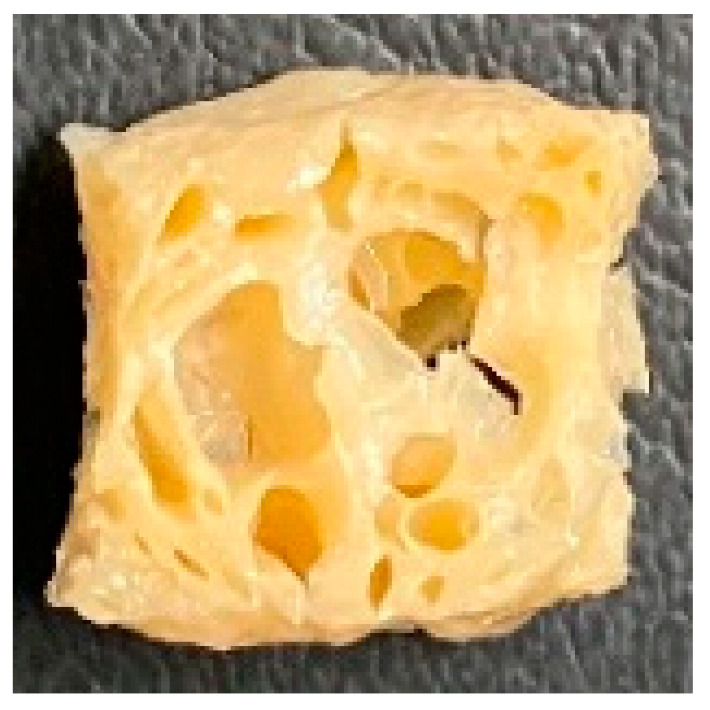	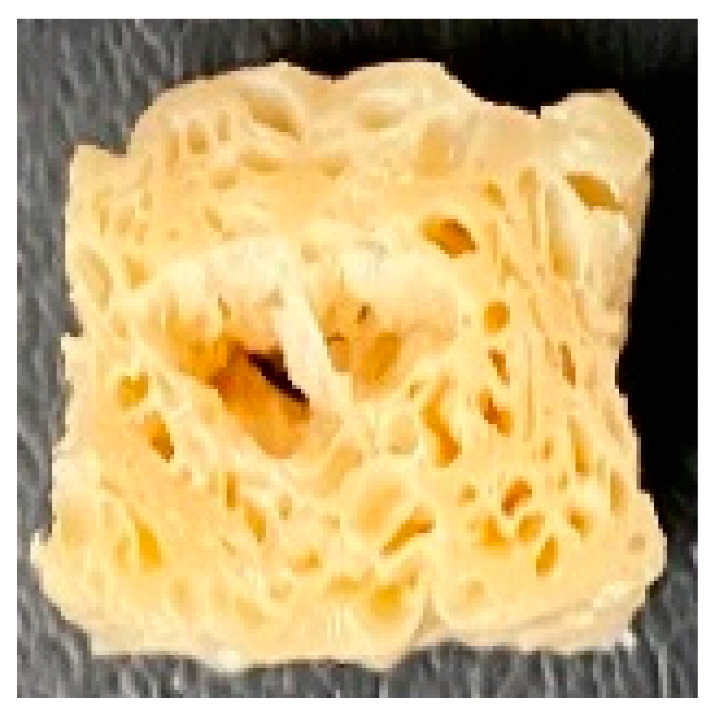	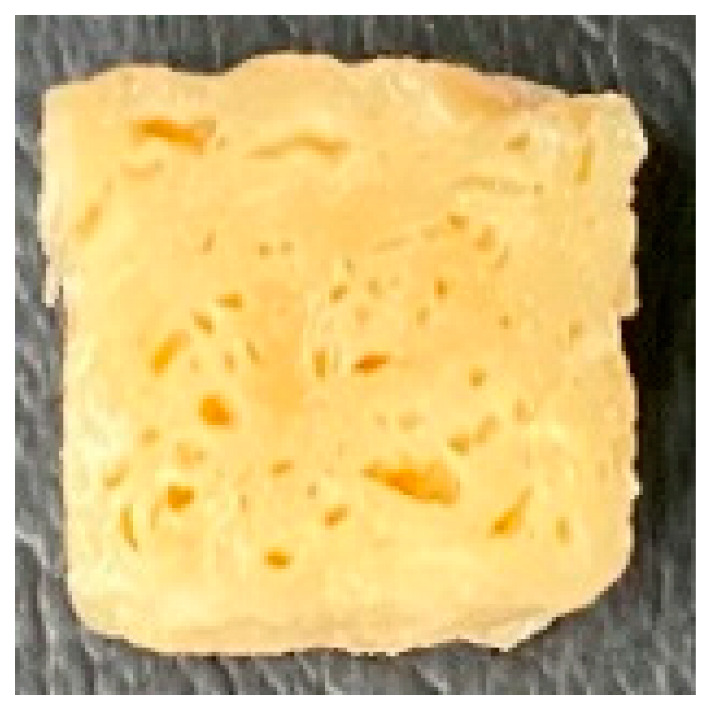	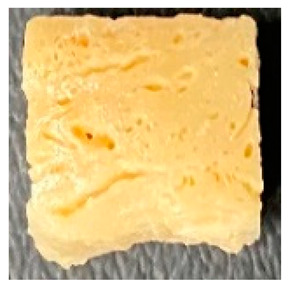		
	Fibrous	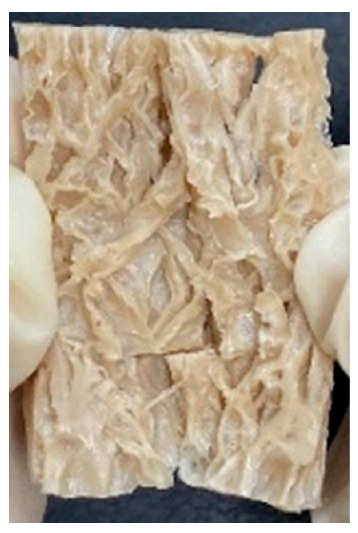	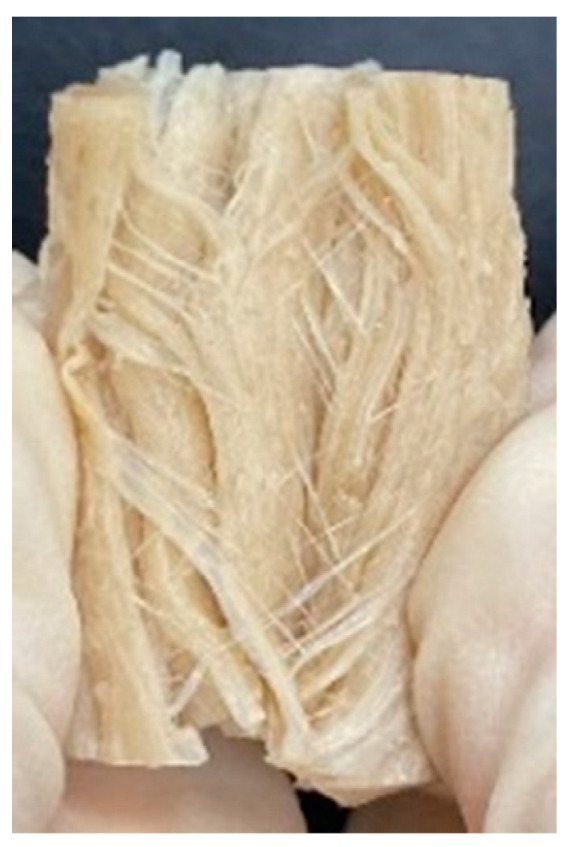	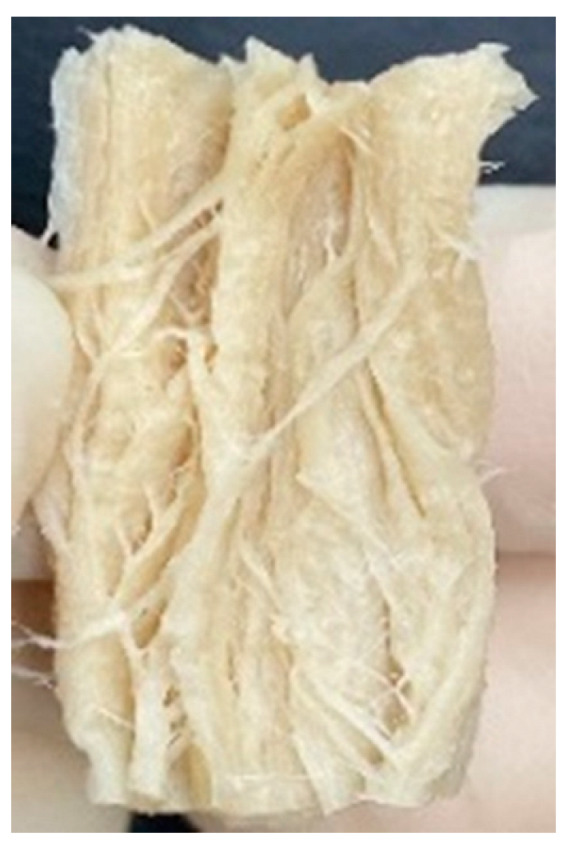	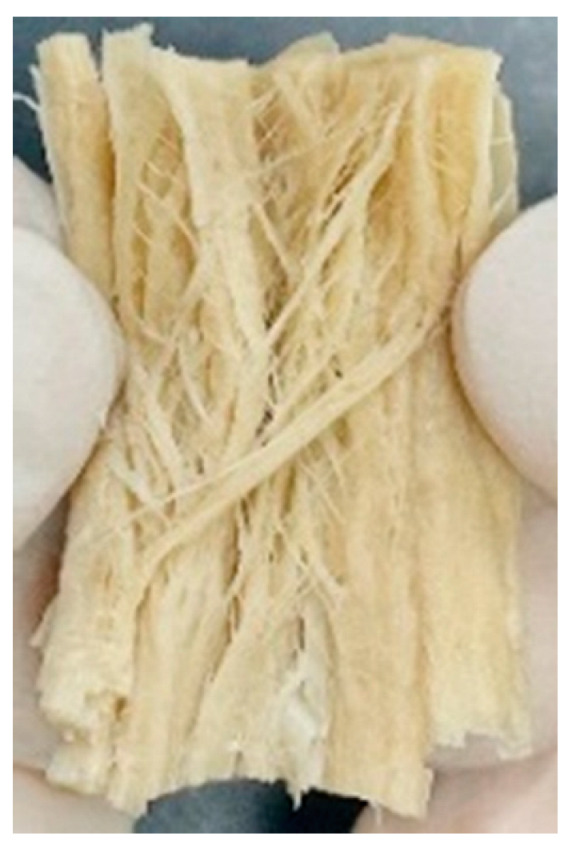		
HMMA	Cross-sectional	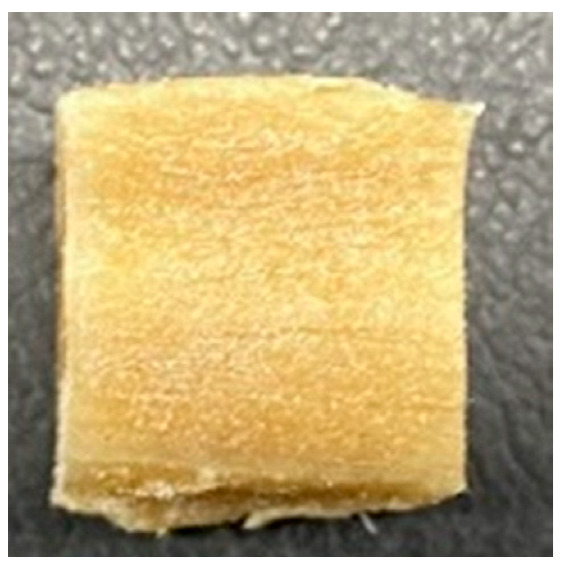	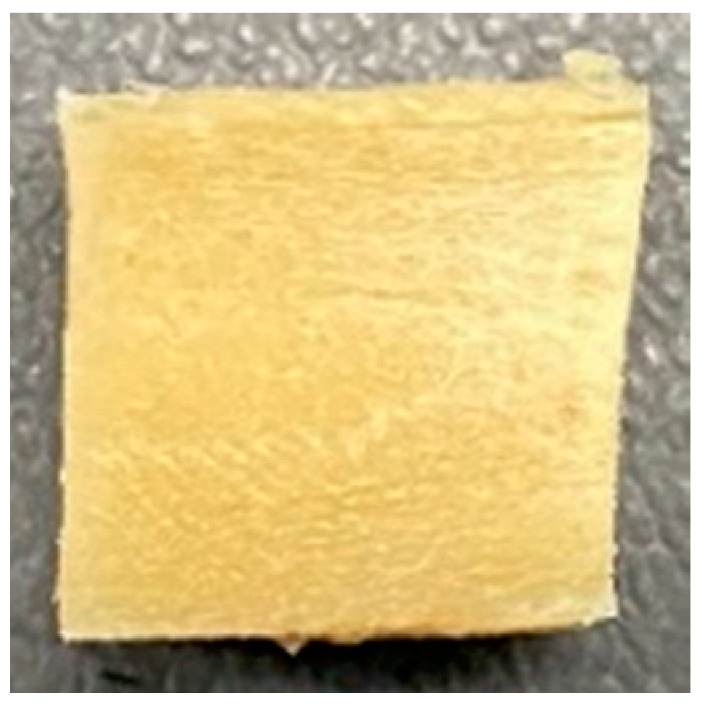	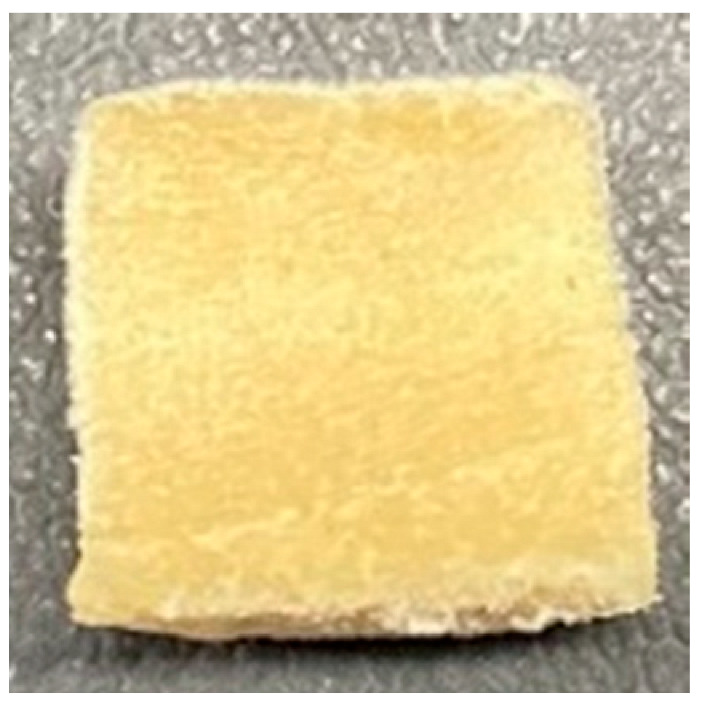	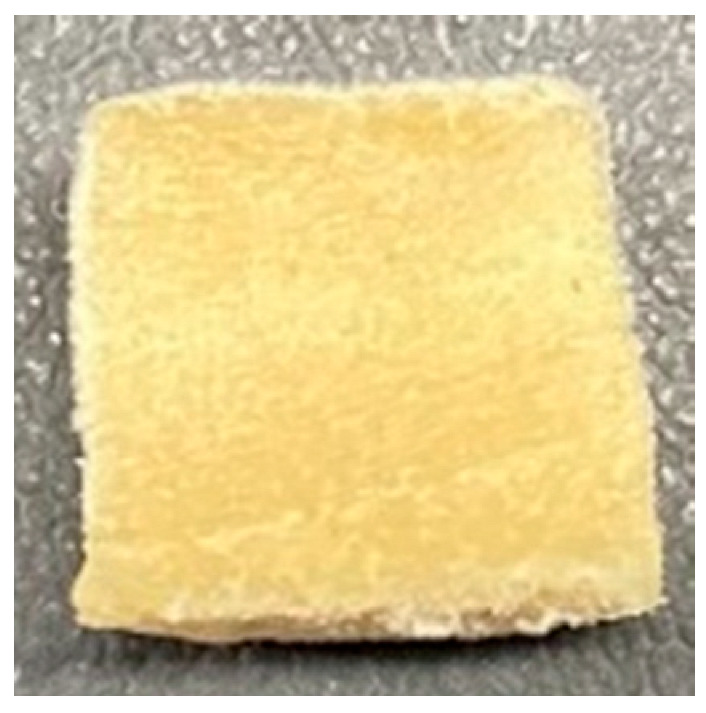	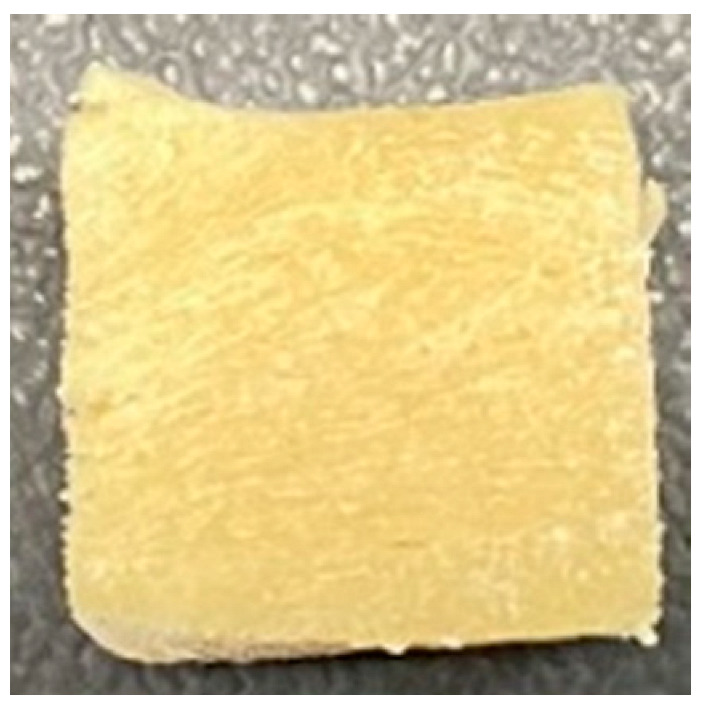	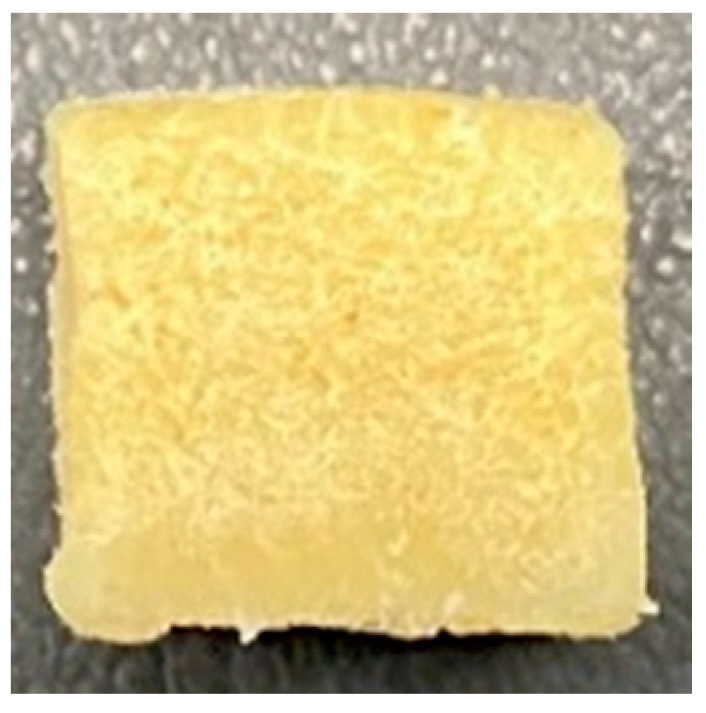
	Fibrous	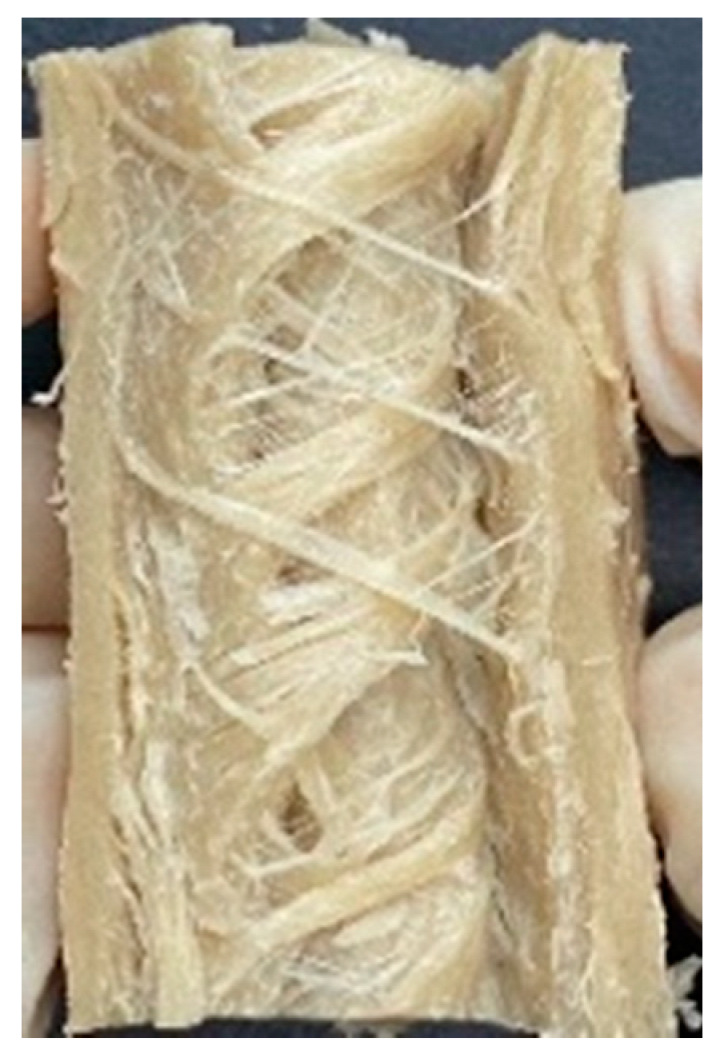	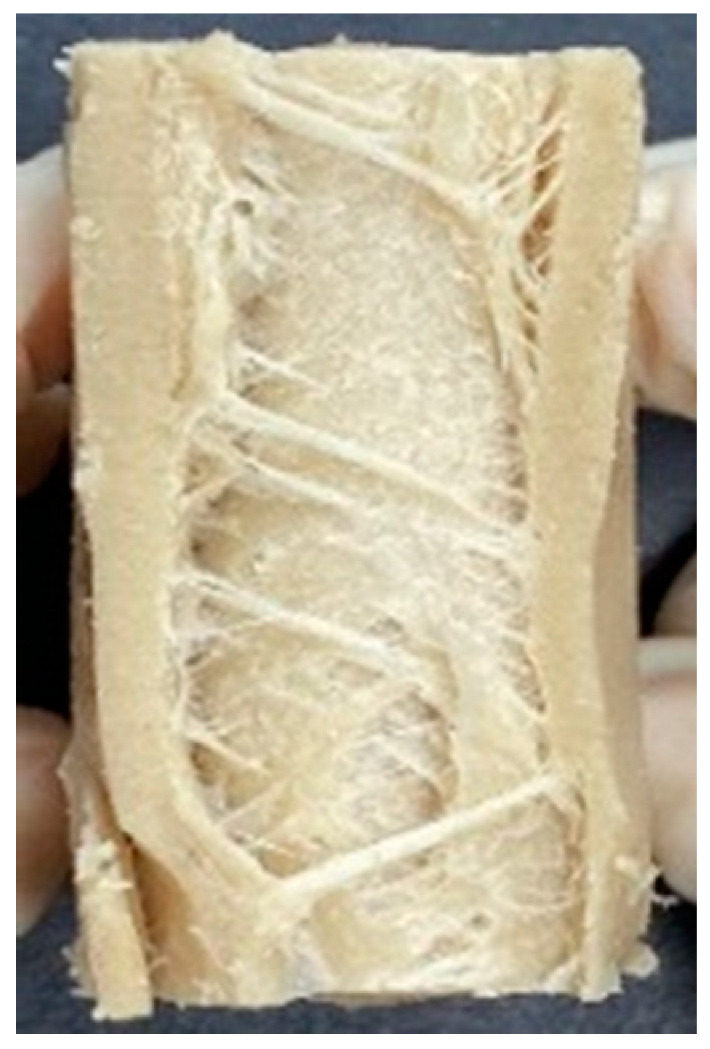	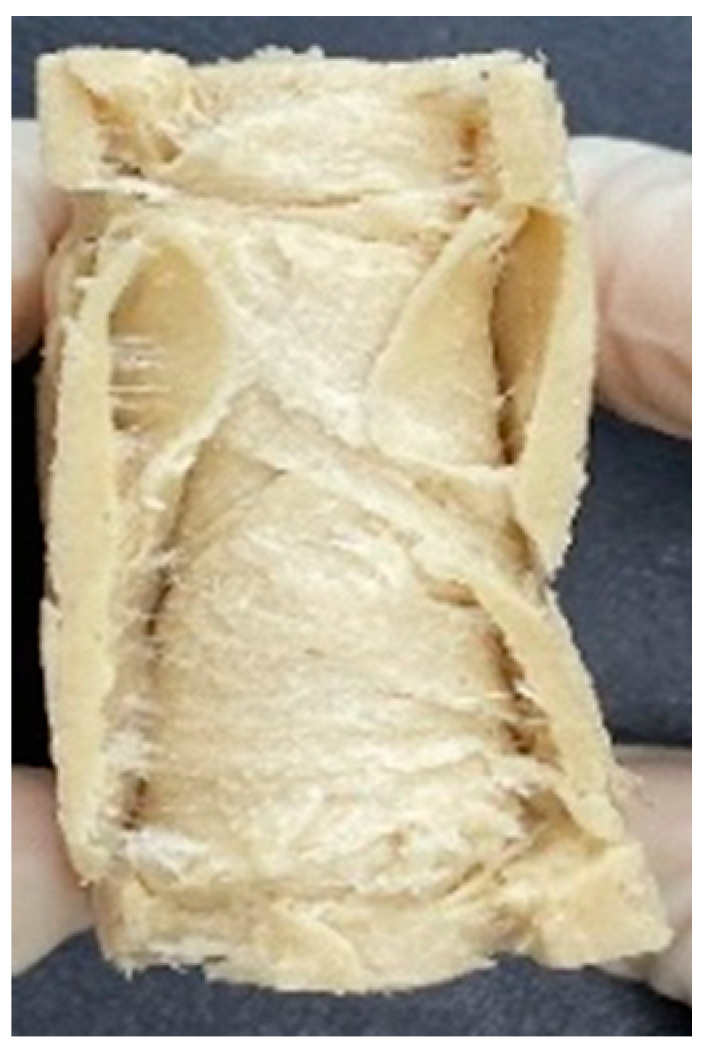	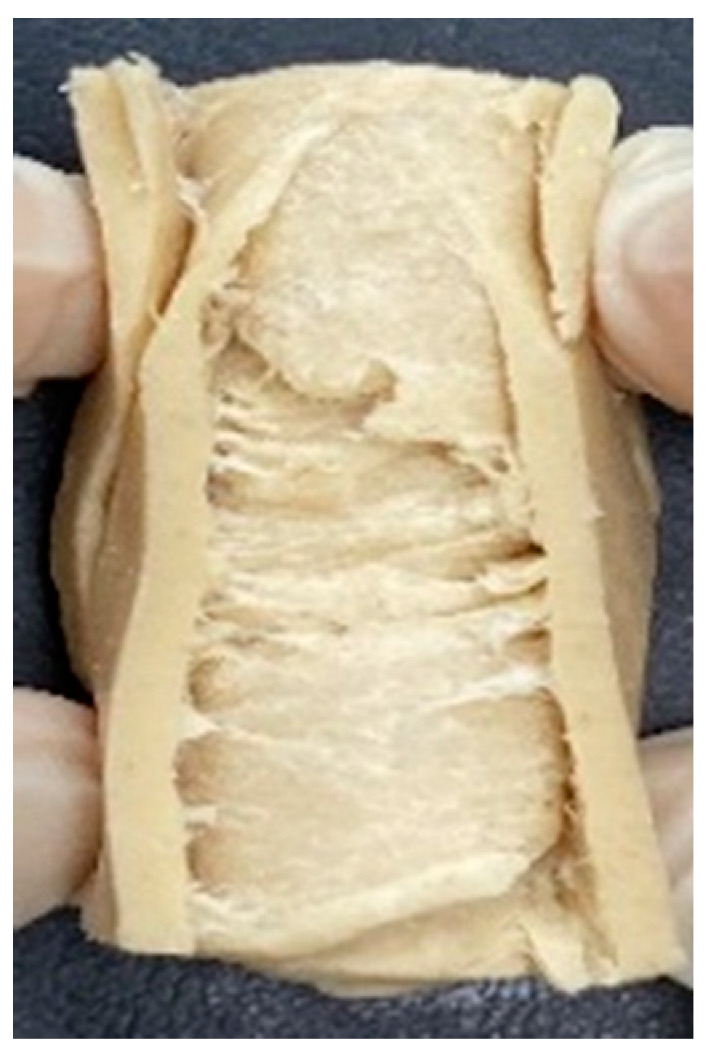	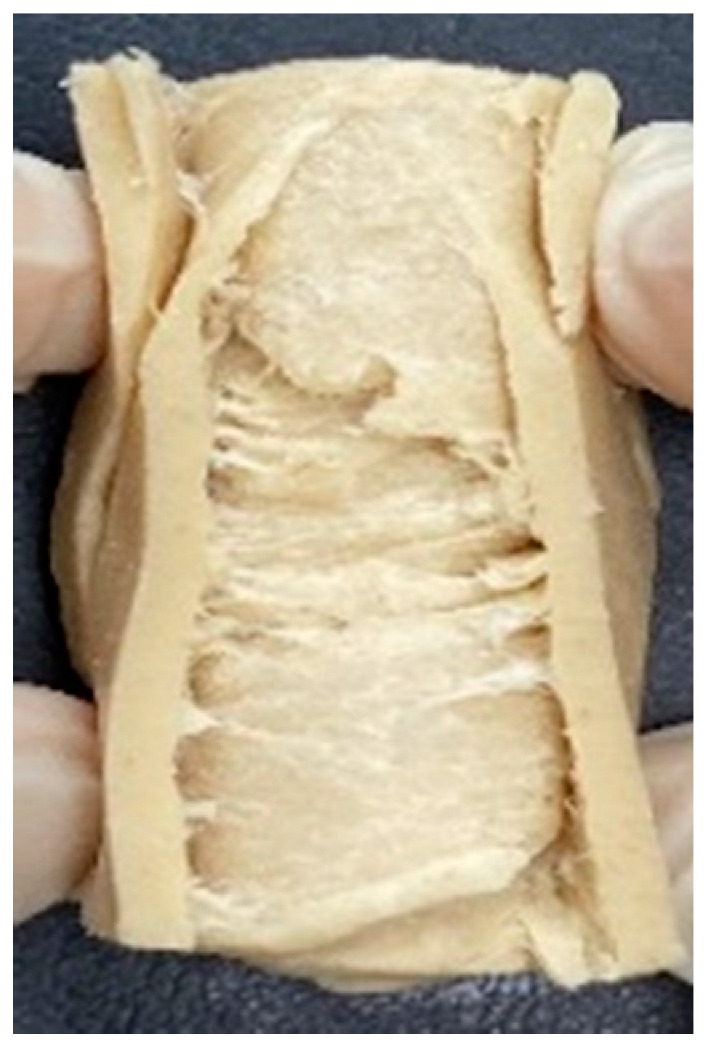	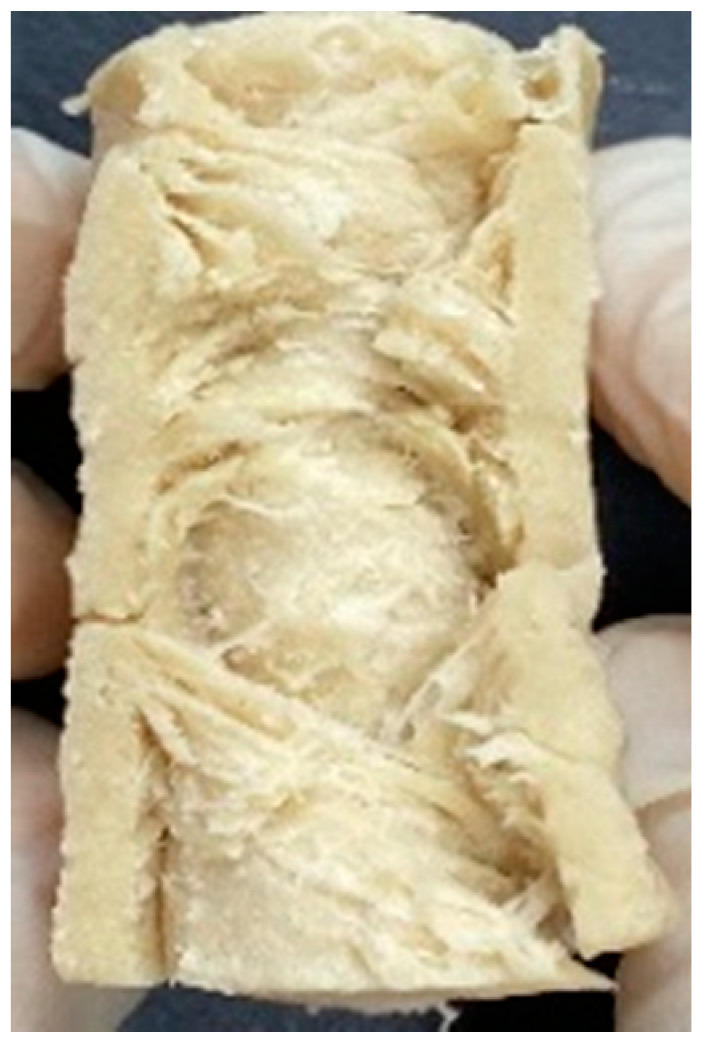

LMMA: low-moisture meat analog; HMMA: high-moisture meat analog.

## Data Availability

Data is contained within the article.
